# Correction: Controlled evaporation-induced phase separation of droplets containing nanogels and salt molecules

**DOI:** 10.1039/d2ra90126a

**Published:** 2022-12-14

**Authors:** Yuandu Hu

**Affiliations:** Departments of Materials Science and Engineering, Department of Physics, School of Physical Science and Engineering, Beijing Jiaotong University Beijing China huyd@bjtu.edu.cn

## Abstract

Correction for ‘Controlled evaporation-induced phase separation of droplets containing nanogels and salt molecules’ by Yuandu Hu *et al.*, *RSC Adv.*, 2022, **12**, 27977–27986, https://doi.org/10.1039/D2RA04585K.

The author regrets that the funding information was incorrectly shown in the acknowledgements section of the original manuscript. The corrected funding acknowledgement is as shown below.

Dr Yuandu Hu is grateful for the Fundamental Research Funds for the Central Universities under the grant number KSRC21006532.

The Royal Society of Chemistry apologises for these errors and any consequent inconvenience to authors and readers.

## Supplementary Material

